# DNA-free genome editing in grapevine using CRISPR/Cas9 ribonucleoprotein complexes followed by protoplast regeneration

**DOI:** 10.1093/hr/uhac240

**Published:** 2022-10-26

**Authors:** Samaneh Najafi, Edoardo Bertini, Erica D’Incà, Marianna Fasoli, Sara Zenoni

**Affiliations:** Department of Biotechnology, University of Verona, 37134 Verona, Italy; Department of Biotechnology, University of Verona, 37134 Verona, Italy; Department of Biotechnology, University of Verona, 37134 Verona, Italy; Department of Biotechnology, University of Verona, 37134 Verona, Italy; Department of Biotechnology, University of Verona, 37134 Verona, Italy

## Abstract

CRISPR/Cas9 genome editing technology can overcome many limitations of traditional breeding, offering enormous potential for crop improvement and food production. Although the direct delivery of Cas9-single guide RNA (sgRNA) ribonucleoprotein (RNP) complexes to grapevine (*Vitis vinifera*) protoplasts has been shown before, the regeneration of edited protoplasts into whole plants has not been reported. Here, we describe an efficient approach to obtain transgene-free edited grapevine plants by the transfection and subsequent regeneration of protoplasts isolated from embryogenic callus. As proof of concept, a single-copy green fluorescent protein reporter gene (*GFP*) in the grapevine cultivar Thompson Seedless was targeted and knocked out by the direct delivery of RNPs to protoplasts. CRISPR/Cas9 activity, guided by two independent sgRNAs, was confirmed by the loss of GFP fluorescence. The regeneration of GFP^−^ protoplasts into whole plants was monitored throughout development, confirming that the edited grapevine plants were comparable in morphology and growth habit to wild-type controls. We report the first highly efficient protocol for DNA-free genome editing in grapevine by the direct delivery of preassembled Cas9-sgRNA RNP complexes into protoplasts, helping to address the regulatory concerns related to genetically modified plants. This technology could encourage the application of genome editing for the genetic improvement of grapevine and other woody crop plants.

## Introduction

Grapevine (*Vitis vinifera* L.) is one of the most widely grown fruit crops in the world, and its fresh and processed fruits, and especially the resulting wines, have a high economic and cultural value [23]. The genetic improvement of crop plants is a key strategy to adapt agricultural production to climate change, higher demands on product quality and quantity, and product differentiation [24, 43, 48]. Conventional breeding is a long established strategy for crop improvement but it can take many years to bring innovative crosses onto the market. The direct transfer of genes and other genetic elements into elite crops produces genetically modified (GM) varieties with desirable features much more rapidly than conventional breeding, but the GM products are hampered by (mostly unconfirmed) health and environmental safety concerns. The benefits of GM technology are therefore limited to a small number of crops [48].

The limitations of conventional breeding and GM technology can be overcome by genome editing, which accelerates basic research and plant breeding by allowing the rapid introduction of targeted mutations [24]. Genome editing technologies favor the production of small insertions or deletions that create gene knockouts, but more sophisticated approaches allow for allele replacement and targeted insertions, which can also be valuable for crop improvement [48]. The CRISPR/Cas9 system (*clustered regular interspaced short palindromic repeats/CRISPR-associated protein9*) is a cutting-edge genome-editing technology that can be usedto introduce mutations without the integration of foreign DNA.In the simplest approach, the endonuclease Cas9 is placed at a specific genomic target region by a single guide RNA (sgRNA)with a matching sequence. The RNA-guided Cas9 protein then introduces a site-specific double-stranded DNA break, which is generally repaired by the error-prone non-homologous end joining pathway to create the insertion/deletion mutation, but in the presence of a donor template can also be resolved cleanly by homology-dependent repair [13]. The direct delivery of Cas9 andsgRNA as a ribonucleoprotein (RNP) complex ensures that no foreign DNA integrates into the genome.

In grapevine, CRISPR/Cas9 technology was initially used toinvestigate gene functions, beginning with the l-idonate dehydrogenase gene (*IdnDH*) in cell suspension cultures of cv. Chardonnayand the phytoene desaturase gene (*PDS*) in somatic embryos of cv. Neo Muscat [25, 31]. This was followed by knocking out the *CCD8* gene, involved in the control of shoot architecture, in embryogenic cells derived from 41B rootstock [30]. The same approach hasbeen used to target genes involved in grapevine disease resistance. Wang *et al*. [42] generated edited lines of cv. ThompsonSeedless with biallelic mutations in the *WRKY52* transcriptionfactor gene, conferring improved resistance to noble rot caused by *Botrytis cinerea*. Sunitha and Rock [35] used CRISPR/Cas9 to edit plants derived from 101–14 rootstock, targeting the *TAS4b* and *MYBA7* genes encoding candidate effectors of Pierce’s disease and grapevine red blotch virus etiology. Furthermore, editing the *MLO3* gene improved resistance to grapevine powdery mildew [40], whereas editing the *PR4b* gene increased susceptibility to *Plasmopara viticola* [15]. CRISPR/Cas9 has also been used to generate multiple alleles of the transcription factor gene *PLATZ1* in a rapid cycling hermaphrodite genotype to demonstrate its involvement in female flower morphology [12]. Most recently, this technology has been used to characterize the role of *EPFL9–1* in the regulation of stomatal density [4] and of *bZIP36* in the control of anthocyanin biosynthesis [38].

Although these studies demonstrated the efficiency of genome editing in grapevine, all were based on the stable integration of genes encoding the CRISPR/Cas9 components using standard gene transfer mediated by *Agrobacterium tumefaciens*, thus creating GM vines. To comply with the mandated absence of foreign DNA in new breeding technologies, two main strategies can be used to develop transgene-free edited plants: (1) the removal of the CRISPR/Cas9 genetic components after editing, and (2) the direct delivery of Cas9-sgRNA RNPs (assembled *in vitro*). T-DNA cassettes can be removed by self-fertilization and segregation in the first generation of offspring, but at the expense of undesirable genetic background changes. Alternatively, T-DNA cassettes can be excised by site-specific recombination, but this leaves a footprint of the recombination site that is likely to be incompatible with current regulations governing GM plants in many countries [6, 7]. The direct delivery of CRISPR/Cas9 RNPs to grapevine was firstly demonstrated in protoplasts of cv. Chardonnay [21], followed by the publication of a stepwise protocol for the design of CRISPR/Cas9 components and their transfer to apple and grapevine protoplasts [27]. However, whole grapevine plants regenerated from edited protoplasts have not been reported thus far.

Recently, a procedure for the regeneration of whole plants from embryogenic callus-derived protoplasts was reported for the Italian varieties Garganega and Sangiovese [3], paving the way toward the development of regeneration protocols for other grapevine varieties. Here we demonstrated the regeneration of transgene-free edited grapevine plants from protoplasts of a transgenic cv. Thompson Seedless line expressing a single-copy green fluorescent protein reporter gene (*GFP*). The protoplasts were transfected with four different RNPs targeting *GFP*, two of which led to the recovery of GFP^−^ mutants that regenerated into whole plants. The regenerated plants showed normal morphology and growth, confirming that RNP delivery by polyethylene glycol (PEG)-mediated transfection to protoplasts is suitable for the efficient generation of transgene-free edited grapevine lines.

## Results

### Generation of somatic embryos overexpressing GFP

**Figure 1:**
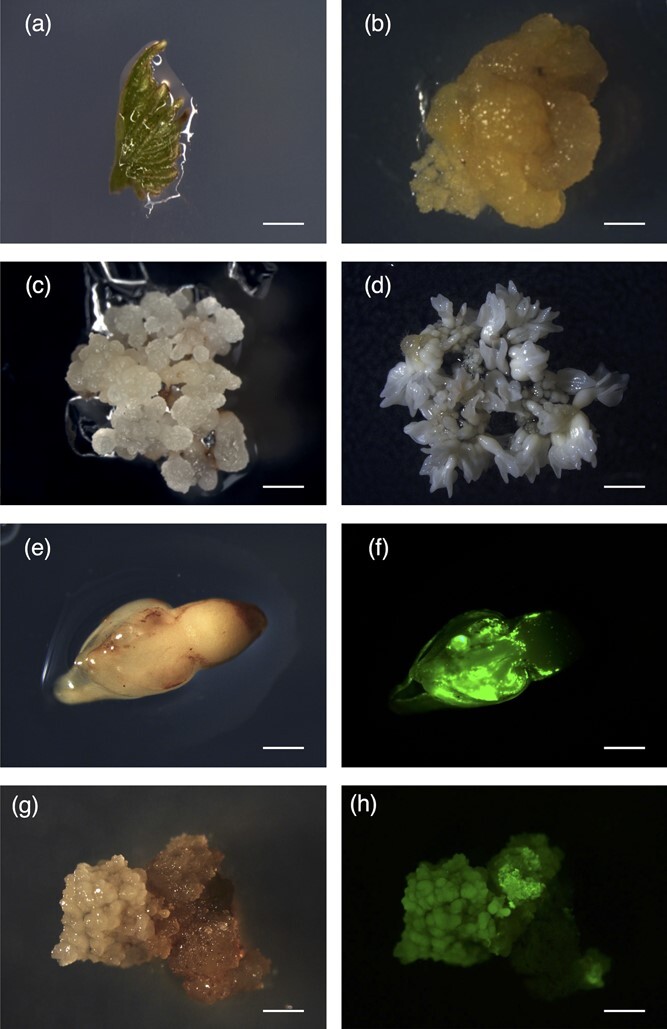
Grapevine embryogenic callus system (cv. Thompson Seedless) and stable transformation with the *GFP* gene. (a) Unopened leaves cultured on initiation medium (bar = 6 mm) produced (b) sectors of embryogenic and non-embryogenic callus (bar = 2 mm). (c) Embryogenic callus proliferated and (d) produced somatic embryos ready for transformation (bars = 1 mm). (e) Somatic embryos after 72 h of co-cultivation with *Agrobacterium tumefaciens* under white light and (f) UV light (bars = 0.5 mm). (g) Embryogenic callus induced from somatic embryos under white light and (h) UV light (bars = 0.75 mm).

We generated a stable grapevine cell line (cv. Thompson Seedless) overexpressing the *GFP* gene to serve as starting material for our genome editing experiments. This was achieved by inducing embryogenic callus from unopened leaf explants after 8 weeks in NB2 medium (Fig. 1a-c). Somatic embryos were regenerated from the callus after 4 weeks in X6 medium (Fig. 1d) and ~ 150 of the embryos at the mid-cotyledonary stage were then co-cultivated with *A. tumefaciens* carrying a GFP overexpression vector (Fig. 1e, f). The explants were transferred to a selective embryogenic callus induction medium to identify transgenic callus material. We found that 12% of the somatic embryos induced embryogenic callus after ~10 weeks, ~83% of which were positive for GFP expression (Fig. 1g, h).

### Isolation of transgenic protoplasts and regeneration into whole plants

**Figure 2:**
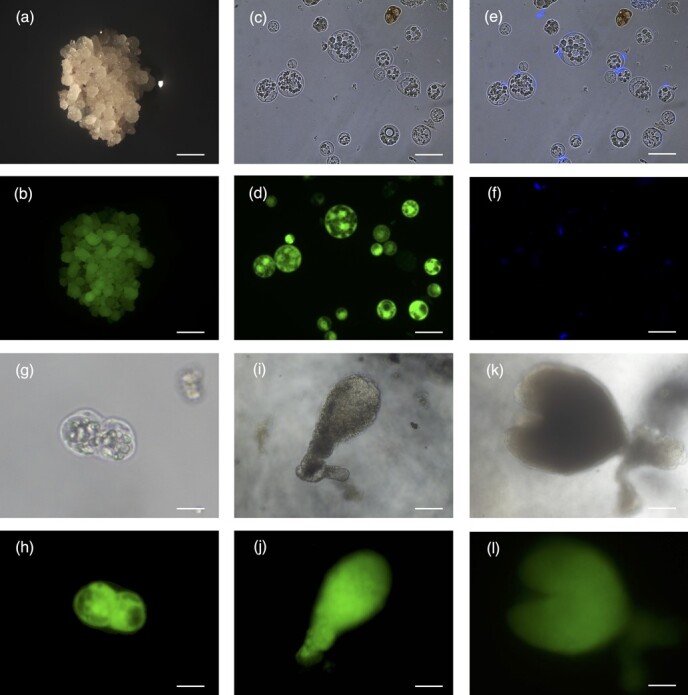
Isolation of grapevine protoplasts (cv. Thompson seedless) overexpressing GFP, and the subsequent regeneration stages. (a) Embryogenic callus overexpressing GFP under white light and (b) UV light (bars = 1 mm). (c) Protoplasts isolated from transgenic callus, viewed under white light and (d) UV light (bars = 20 μm). (e) Protoplasts stained with Fluorescent Brightener 28 under white light and (f) UV light (bars = 20 μm). (g) The first protoplast cell division occurred after 3 days, viewed under white light and (h) UV light (bars = 30 μm). (i) Somatic embryos at the globular stage of embryo development under white light and (j) UV light (bars = 70 μm). (k) Somatic embryos at the heart stage of embryo development under white light and (l) UV light (bars = 70 μm).

Protoplasts were isolated from transgenic embryogenic callus expressing GFP (Fig. 2a, b) with a final yield of up to 4 × 10^7^ protoplasts per gram of callus material. The isolated protoplasts were analyzed to determine their integrity and morphology (Fig. 2c, d) and to evaluate the efficiency of cell wall digestion by staining with Fluorescent Brightener 28 (Fig. 2e, f). Most protoplasts displayed the correct circular shape immediately after isolation, indicating that the majority of the cell walls were digested. Furthermore, all protoplasts showed green fluorescence derived from *GFP* expression. Isolated protoplasts were cultivated at a density of 1 × 10^5^ protoplasts/mL using the disc-culture method, and the GFP signal was monitored weekly. The first protoplast cell division occurred after 3 days (Fig. 2g, h) and further divisions occurred over the next 3 weeks. Microcolonies were observed ~20 days after protoplast isolation. After 30–40 days, somatic embryos began to form showing the typical globular (Fig. 2i, j) and heart stages of embryo development (Fig. 2k, l).

**Figure 3:**
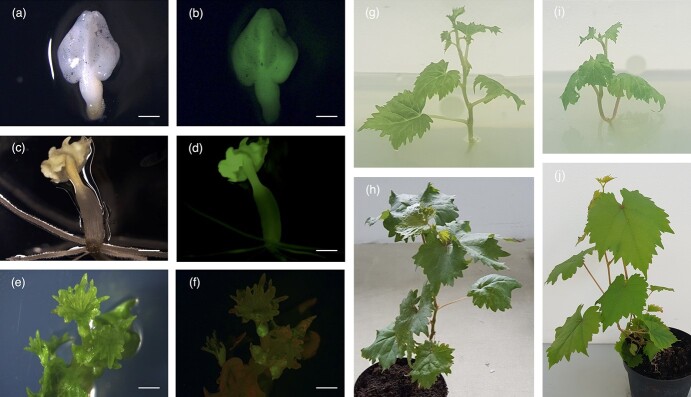
Regeneration of plants from protoplasts overexpressing GFP. (a) Mature cotyledonary somatic embryos under white light and (b) UV light (bars = 0.5 mm). (c) Germinated somatic embryos under white light and (d) UV light (bars = 0.8 mm). (e) Apical young leaves of a regenerated plantlet under white light and (f) UV light (bars = 1 mm). (g) Transgenic plantlet regenerated *in vitro*. (h) Regenerated whole transgenic plant in the greenhouse. (i) Wild-type plantlet regenerated *in vitro*. (j) Wild-type plant in the greenhouse.

We recovered 74 mature cotyledonary embryos 2 months after the initiation of protoplast culture and plated them on germination medium (Fig. 3a, b). All mature cotyledonary embryos produced a GFP signal, confirming that 100% of the isolated protoplasts were transgenic. After 1 month, 48 somatic embryos were selected by assessing their morphology and GFP fluorescence, and were transferred to shoot-inducing medium (Fig. 3c, d).

To identify the most efficient shoot-induction conditions, we evaluated four types of media by adding the synthetic plant hormone 6-benzylaminopurine (BAP) to the basal media C2D and MG1. The four conditions were C2D with no BAP, C2D plus 4 μM BAP (C2D + 4B), MG1 with no BAP, and MG1 plus 10 μM BAP (MG1 + 10B). We plated 12 embryos on each medium and, when plantlets grew within 3–5 weeks, we monitored the GFP fluorescence (Fig. 3e, f). C2D + 4B was the most efficient medium for inducing shoots, achieving a regeneration frequency of 75% (Table 1, Fig. S1).

**Table 1:** Somatic embryogenesis and plant regeneration from grapevine (cv. Thompson Seedless) protoplasts overexpressing GFP

Cultivated Protoplast	N° Mature Embroys	N° Germinated Embroys	N° Germinated Embroys			N° Whole Plants		
			C2D	C2D + 4b	MG1	MG1 + 10B	MSN (from C2D)	RIM (from MG1)
12x10^5	74	48	4/12 (33%)	9/12 (75%)	8/12 (66%)	3/12 (25%)	12/13 (91%)	10/11 (91%)

To promote root development, shoots germinated in CD2 or C2D + 4B were transferred to MSN, and those germinated in MG1 or MG1 + 10B were transferred to RIM. After 1 month, all plantlets except one in each medium developed into whole plants with expanded leaves and roots (Fig. 3g). The protoplast regeneration efficiency from mature cotyledonary embryos to whole plants was therefore ~30% (22/74). The presence of the *GFP* transgene was verified by PCR (Fig. S2). The regenerated plants were finally transferred to the greenhouse and, after a period of acclimation, showed normal growth and morphology compared to wild-type plants (Fig. 3h-j).

### Knockout of the *GFP* gene by the direct delivery of CRISPR/Cas9 RNPs

**Figure 4:**
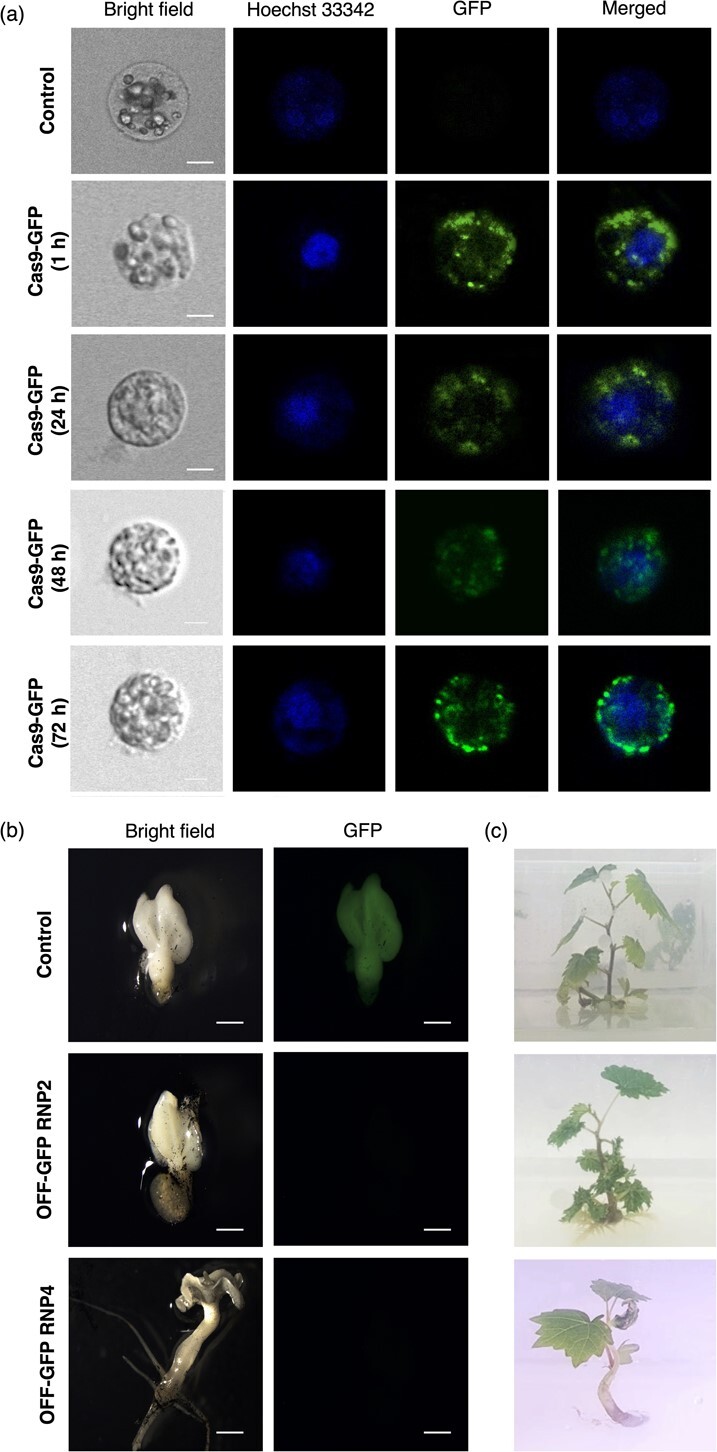
Nuclear localization of Cas9-GFP in grapevine (cv. Thompson Seedless) protoplasts and somatic embryos, and the regeneration of plantlets from protoplasts transfected with RNP2 and RNP4. (a) Nuclear localization of Cas9-GFP in protoplasts 1, 24, 48 and 72 h post-transfection, viewed under white light (bright field), with nuclear staining (Hoechst 33342), under UV light (GFP) and a merged view of the Hoechst 33342 and GFP images (merged), compared to a PEG-only transfection control (bars = 5 μm). (b) Germinated somatic embryos regenerated from protoplasts overexpressing GFP (control), from protoplasts transfected with RNP2 and lacking GFP activity (OFF-GFP RNP2) and from protoplasts transfected with RNP4 and lacking GFP activity (OFF-GFP RNP4) under white light (bright field) and UV light (GFP) (bars = 0.8 mm). (c) Phenotype of plantlets regenerated *in vitro* from PEG-transfected protoplasts (control) and from those transfected with RNP2 (OFF-GFP RNP2) and RNP4 (OFF-GFP RNP4).

We transfected 2 × 10^5^ wild-type cv. Thompson Seedless protoplasts with 60 μg of a Cas9-GFP fusion protein and monitored the emission of fluorescence by confocal microscopy for 72 h (Fig. 4a). GFP fluorescence was clearly detected in both the cytosol and nuclei of the transfected protoplasts from 1 to 72 h post-transfection, suggesting that Cas9 can be introduced into protoplasts efficiently by PEG-mediated transfection and maintains its ability to reach the nucleus. We did not detect any fluorescence in control transfections with PEG alone. We estimated that the efficiency of protoplast transfection was ~17% by comparing the number of fluorescent and total transfected protoplasts 1 h post-transfection (Fig. S3).

We then used the same protocol to deliver Cas9-sgRNA RNPs targeting the *GFP* gene to transgenic protoplasts expressing GFP. Four target sites were identified in the 5′ region of the *GFP* gene using the online tools CRISPOR (http://crispor.org/) and CRISPR RGEN (http://www.rgenome.net/), which take into account the GC content and any putative off-target sites (Fig. S4). The Cas9-sgRNA RNPs were named RNP1, RNP2, RNP3 and RNP4. Isolated protoplasts expressing GFP (6.5 × 10^7^ protoplasts/g) were transfected with Cas9 and sgRNA at a 1:1 weight ratio (60 μg each component) or with PEG only as a transfection control, followed immediately by cultivation using the disc-culture method. The protoplasts were monitored every week for cell division, embryogenesis and GFP fluorescence. After 2 months, regenerated cotyledonary somatic embryos were transferred to germination medium for 4 weeks. We obtained 42 mature cotyledonary embryos transfected with RNP1, 35 with RNP2, 37 with RNP3, 29 with RNP4, and 27 from the transfection control. We identified two embryos (one transfected with RNP2 and one with RNP4) lacking GFP fluorescence (GFP^−^), providing initial evidence of the success of targeted mutations caused by RNP activity (Fig. 4b). The embryos obtained from transfection with RNP1 and RNP3 retained their GFP signal and were excluded from further analysis. After 1 month, we obtained 23 out of 35 and 23 out of 29 well-developed germinated somatic embryos transfected with RNP2 and RNP4, respectively (including the GFP^−^ RNP2 and RNP4 embryos), and 16 out of 27 for the control transfection. All somatic embryos were transferred to C2D + 4B regeneration medium for 4 weeks, finally yielding 8/23, 10/23 and 5/16 plantlets from the transfections with RNP2, RNP4 and the control, respectively. These included one GFP^−^ plant among the eight transfected with RNP2 and one GFP^−^ plant among the 10 transfected with RNP4, both of which showed a normal morphological phenotype and growth habit compared to control plants (Fig. 4c). Overall these data showed that the regeneration efficiency of transfected protoplasts from mature cotyledonary embryos to whole plants was 23% for RNP2 and 34% for RNP4, and was not affected by the transfection process or the time required to obtain whole plants.

### Evaluation of *GFP* mutations

**Figure 5:**
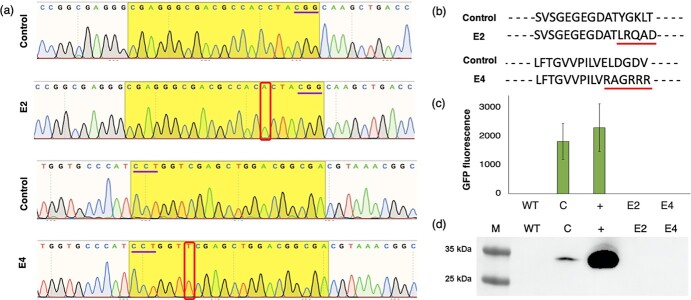
Analysis of GFP mutations in edited plants. (a) Adenine and thymidine insertions (red boxes) in plants regenerated from protoplasts transfected with RNP2 (E2) and RNP4 (E4), both lacking GFP activity, compared to plants regenerated from PEG-transfected protoplasts (control). The PAM sequence is underlined in violet. (b) GFP amino acid sequence frameshift in plants E2 and E4 compared to control. (c) GFP fluorescence intensity based on total protein extracted from plants E2 and E4 compared to wild-type (WT) plants (lacking GFP) and control (C) transgenic plants expressing GFP. The (+) lane is an additional positive control of total protein extracted from GFP-overexpressing tobacco plants. Data are means ± SE (n = 3). (d) Western blots of total protein extracted from E2 and E4 leaves compared to WT and C samples, probed with an anti-GFP polyclonal antibody. The same polyclonal antibody is used here as an additional positive control for the detection step. The anticipated molecular weight of GFP is 27 kDa. M, marker (PageRuler Pre-stained Protein Ladder, 10—180 kDa; Thermo Fisher Scientific).

To verify whether the CRISPR/Cas9 system induced mutations in the *GFP* gene, we extracted genomic DNA from OFF-GFP RNP2 and OFF-GFP RNP4 leaves and confirmed the presence of a single-copy *GFP* transgene by droplet digital PCR (ddPCR) analysis (Fig. S5). We then sequenced the genomic DNA and found that the *GFP* gene was edited in both plants, thus excluding the possibility that GFP^−^ plants were regenerated from wild-type embryos. OFF-GFP RNP2 (E2) featured an adenine insertion three nucleotides upstream of the PAM sequence, whereas OFF-GFP RNP4 (E4) featured a thymidine insertion three nucleotides downstream of the PAM sequence (Fig. 5a). The remaining regenerated plants, seven recovered following protoplast transfection with RNP2 and nine with RNP4, were also sequenced, and no mutations were found in the *GFP* gene (data not shown). The insertions in E2 and E4 caused frameshifts in the *GFP* coding sequence (Fig. 5b) that abolished the GFP signal (Fig. 5c). This reflected the complete loss of GFP in both E2 and E4, as confirmed by western blot analysis with an anti-GFP antibody (Fig. 5d).

## Discussion

CRISPR/Cas9 is a powerful genome editing technology that has been widely adopted in plants due to its versatility and compatibility with DNA-free editing approaches. It has been applied in many species, including tomato [28], wheat [18, 47], rice [45], petunia [34], maize [36] and grapevine [31]. However, although research in grapevine has focused on the direct delivery of CRISPR/Cas9 components without stable transformation, such as the transfection of protoplasts [21, 27], and on the regeneration of whole plants from isolated protoplasts [3], these two procedures have yet to be combined.

We have achieved DNA-free genome editing in grapevine by the direct introduction of RNPs into protoplasts, followed by the regeneration of transfected protoplasts into whole plants. We demonstrated proof of concept by delivering RNPs into protoplasts isolated from embryogenic callus of a transgenic cv. Thompson Seedless line expressing GFP, and confirmed successful editing by demonstrating the absence of GFP activity and the presence of single-nucleotide insertion mutations in the *GFP* transgene.

**Figure 6:**
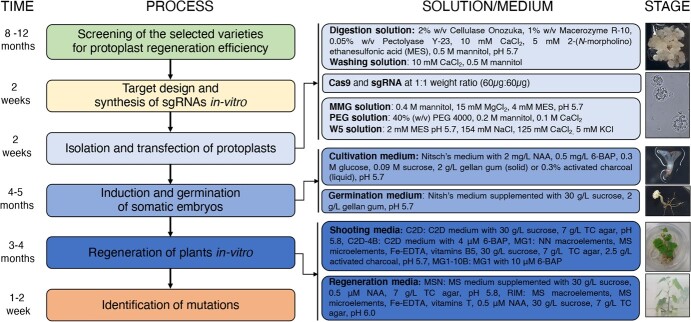
Flow chart of CRISPR/Cas9-mediated gene editing in grapevine, including a step-by-step protocol based on the direct delivery of RNPs to protoplasts. The entire procedure takes ~18 months.

The entire experiment cycle lasted ~18 months from the analysis of protoplast regeneration to the identification of target site mutations in the regenerated plants (Fig. 6). Major hurdles included the formation of embryogenic callus from grapevine tissue because the efficiency of somatic embryogenesis in grapevine is strictly varietal-specific, and the regeneration of whole plants from protoplasts after transfection, which required the time-consuming optimization of media and cultivation parameters [3]. Our approach is based on targeting single cells (protoplasts) so that each one can regenerate independently into a potentially edited whole plant, thus limiting the occurrence of genetic chimeras [29, 39]. Unlike the organogenesis pathway, regenerating plants from protoplasts via somatic embryogenesis ensures the genetic homogeneity of the final product.

An additional challenge we addressed was the coupling of efficient protoplast isolation and regeneration procedures with the transfection of RNPs into protoplasts, adapting the protocols for grapevine. Although we were successful, the technical aspects may need further optimization for additional economically important élite cultivars that are particularly recalcitrant to gene transfer and/or regeneration [10].

Somaclonal variation is frequently observed among grapevines regenerated via somatic embryogenesis, and this can lead to undesirable traits such as chlorophyll deficiency and aberrant leaf and flower development [22]. We observed no overt phenotypic changes, and the growth and development of our edited plants were comparable to wild-type controls, indicating the absence of disruptive somaclonal variations under the conditions we used. This work was intended as a proof of concept to demonstrate the possibility of regenerating transgene-free edited grapevines from transfected protoplasts using a simple visual screening approach based on GFP. Targeting the *GFP* reporter gene in a transgenic line allowed us to identify targeted mutants and assess the efficiency of mutagenesis at the protein activity level. The *GFP* target region was selected based on recommendations to design multiple targets for each single gene, prioritizing areas with a high GC content (50–70%) because these can significantly improve the mutation efficiency [20, 30, 42, 46]. In agreement with these recommendations, among the four targets designed for the *GFP* gene, we detected mutations at the T2 and T4 sites that indeed feature the highest GC content (Fig. S4).

The CRISPR/Cas9 system typically generates short insertions or deletions [26, 30], whereas we observed only single-nucleotide insertions in the *GFP* gene (Fig. 5). In contrast, a previous study using the same grapevine variety led to the recovery of predominantly short deletions [42]. This may reflect the different approaches used in each study, namely the direct delivery of RNPs targeting a single gene in our study compared to the conventional stable overexpression of CRISPR/Cas9 components targeting multiple sites in the previous study [42].

The combination of molecular biology and tissue culture/propagation parameters we adopted resulted in the successful regeneration of two OFF-GFP grapevine plants (E2 and E4) from a total of eight plants originating from RNP2 protoplasts and 10 from RNP4 protoplasts. This corresponds to an overall editing efficiency of ~11%. We also demonstrated that PEG-mediated transfection does not significantly disrupt grapevine regeneration. A recent preprint reported the regeneration of transgene-free edited grapevine plants from protoplasts of cv. Crimson Seedless, confirming the presence of mutations in the *DMR6–2* gene [33]. Although the methods were not reported in great detail, we found some key differences between the regeneration protocols in the two studies (Table S2). In detail, the protoplast isolation step differs in both the enzymes concentration in the digestion solution and the incubation time, as well as the preparation of RNP components prior PEG transfection and the protoplasts cultivation method were adjusted (Table S2). Seven of the eight plants tested were found to carry *DMR6–2* homozygous mutations, but the authors did not state the total number of regenerated plants and did not describe the plant regeneration procedure, thus making it impossible to calculate the regeneration and editing efficiencies [33]. It is also worth mentioning that our results were obtained using a 1:1 weight ratio of Cas9 and sgRNA for PEG-mediated transfection, offering a further opportunity for optimization by testing different Cas9:sgRNA ratios [14, 19, 42].

In conclusion, we have developed a grapevine genome-editing method for functional research and molecular breeding based on the DNA-free delivery of CRISPR/Cas9 components to protoplasts, which subsequently regenerate into plants that lack exogenous DNA sequences or any footprints of the procedure. Conventional CRISPR/Cas9 genome editing involves the delivery of Cas9 and sgRNA transgenes, which are typically excised by site-specific recombination, leaving footprints of the recombination sites, or they are removed by outcrossing, which changes the genetic background [7]. Alternative systems enabling the direct delivery of CRISPR/Cas9 components to plant cells include replicon-based transient delivery, which is applied to embryogenic callus and involves conventional co-cultivation with *A. tumefaciens* or particle bombardment, potentially leading to the regeneration of chimeric plants [2, 41]. In our approach, somatic embryos were regenerated from single protoplasts, avoiding the callus phase and thereby strongly limiting the likelihood of chimeras. The possibility that plants could be derived from more than one protoplast, resulting in chimeras containing edited and non-edited sectors or chimeras containing two edited sectors with different mutations, was excluded by our observation that all regenerated embryos uniformly expressed (or did not express) GFP and by the analysis of our sequencing data. The ability to obtain transgene-free edited grapevine plants by the direct delivery of preassembled Cas9-sgRNA RNPs to protoplasts may address regulatory concerns related to GM plants. In this context, our new approach could be perceived positively by policymakers and regulators, encouraging the use of genome editing for the genetic improvement of grapevine and other woody and herbaceous crops.

## Materials and methods

### Embryogenic cultures

Embryogenic cultures of cv. Thompson Seedless were generated from the unopened leaves of plantlets grown *in vitro*, 5–6 weeks of age [8]. Five unopened leaves were placed adaxial side down on NB2 medium (Table S1) and incubated in darkness at 27°C for 6–8 weeks. The resulting embryogenic callus was placed into C1^P^ medium (Table S1). The pH was adjusted to 5.8 with KOH after incorporating 5 g/L Phytagel (Sigma-Aldrich, St Louis, MO, USA) as previously described [11]. The callus was maintained as stated above and subcultured at 4-week intervals. For the regeneration of somatic embryos, embryogenic callus was transferred from C1^P^ medium to X6 medium (Table S1; [16]). The embryos were maintained on the same medium for 4 weeks.

### Transformation

The binary vector pEGB3α1-TNOS::NPTII::PNOS-SF-35S::GFP::TNOS-SF was assembled using the Golden Braid 2.0 (GB 2.0) system [32] and was introduced into *A. tumefaciens* strain EHA105 by electroporation. Somatic embryos (cv. Thompson Seedless) were transfromed as previously described by Li *et al*. [16] with minor modifications. Briefly, after inoculation, the embryos were co-cultivated for 72 h before transfer to liquid DMcc medium (Table S1) at 26°C for 24 h on a rotary shaker (110 rpm). The liquid medium was withdrawn and replaced with the same amount of fresh selection DMcck50 medium (Table S1) for the next 48 h. Finally, the embryos were washed with liquid selective DM medium and transferred to solid selective DM medium (Table S1) to induce embryogenic callus. DM medium was modified based on the DKW medium developed by [9] (Table S1). For liquid DM medium, the TC agar was omitted. We placed 30–35 somatic embryos on each culture plate and incubated them in darkness at 26°C for 60 days for callus induction. Transgenic GFP^+^ embryogenic callus picked under a stereomicroscope was transferred to C1^P^cck70 medium (Table S1) for long-term maintenance.

### Protoplast isolation and cultivation

Protoplasts were isolated from wild-type (cv. Thompson Seedless) and GFP^+^ transgenic embryogenic callus as previously described [3, 49]. After 7–10 days in C1^P^ medium of subculture the embryogenic callus was incubated for 5–6 h on a rotating shaker with 10 mL of digestion solution (Table S1) per gram. After filtration through a 60-μm nylon mesh, protoplasts were washed twice with 10 mM CaCl_2_ plus 0.5 M mannitol. Protoplasts viability was determined by staining with 0.5 mg/mL fluorescein diacetate (FDA; Sigma-Aldrich) under UV light. To check for cell wall digestion, wild-type and transgenic protoplasts were stained with 2 μM Fluorescent Brightener 28 (Santa Cruz Biotechnology, Dallas, TX, USA) and visualized under UV light. Transgenic protoplasts at a density of 1 × 10^5^ protoplasts/mL were resuspended in solidifying disc-culture (DC) Nitsch’s medium (Table S1) and 1–5 drops were collected in Petri dishes. When the medium had solidified, liquid DC Nitsch’s medium with 0.3% activated charcoal was added as a reservoir. The protoplast cultures were incubated in darkness at 27°C. Protoplasts were visualized using a DM2500 microscope (Leica Microsystems, Wetzlar, Germany) equipped with UV lamp and filter sets composed of an excitation filter (470/40 nm) and a barrier filter (525/50 nm) for FDA and an excitation filter (340–380 nm) and a barrier filter (LP 425) for Fluorescent Brightener 28. Cultivated transgenic protoplasts and somatic embryo stages were analyzed using an Olympus IX70 microscope equipped with UV lamp and filter sets composed of an excitation filter (470/40 nm) and an emission filter (520 nm).

### Germination of somatic embryos and plant regeneration

After 2–3 months, protoplast-derived mature cotyledonary somatic embryos were moved to Nitsh’s medium supplemented with 30 g/L sucrose and 2 g/L of gellan gum for 4–5 weeks in the dark. Well-developed germinated somatic embryos were transferred into four different types of shooting media: C2D medium supplemented with 30 g/L sucrose, pH 5.8; C2D-4B medium (Table S1; [17]); MG1 (Table S1; [37]); and MG-10B (MG1 medium with 10 μM BAP). Each medium was supplemented with 7 g/L TC agar. Plantlets were incubated for 5–6 weeks with a 16-h photoperiod and the resulting plantlets were transferred to MSN (Table S1; [17]) or RIM (Table S1; [37]) for root induction and whole plant regeneration.

### Cas9 proteins, sgRNA design and *in vitro* synthesis

We used a Cas9-GFP fusion protein (Sigma-Aldrich) for the transfection of wild-type protoplasts and eSpCas9 (Sigma-Aldrich) for the gene editing experiments in transgenic protoplasts. Both versions of Cas9 were translocated to the nucleus following transfection. The sgRNAs were designed using the web tools CRISPOR (http://crispor.org/; [5]) and RGEN Tools (http://rgenome.net/; [1]). The best sgRNA was selected according to scores that evaluate potential off-targets in the grapevine genome and predict on-target activity. The sgRNAs were synthesized using the GeneArt Precision gRNA Synthesis Kit (Invitrogen, Thermo Fisher Scientific, Waltham, MA, USA) according to the manufacturer’s instructions.

### Protoplast transfection and cultivation

Wild-type protoplasts were transfected with Cas9-GFP to confirm nuclear localization, whereas transgenic protoplasts were transfected with RNPs comprising equal amounts by weight of sgRNA (60 μg) and eSpCas9 (60 μg). The components were incubated for 10 min at room temperature in the dark before protoplast transfection, which was carried out as described by Woo *et al*. [44] and Osakabe *et al*. [27] with minor modifications. Briefly, a mixture of 2 × 10^5^ protoplasts suspended in 200 μL MMG (Table S1) was mixed with 20–30 μL of the RNP complex and 200 μL 40% (w/v) PEG 4000 in 0.2 M mannitol and 0.1 M CaCl_2_ before incubation for 20 min at room temperature in darkness. The protoplasts were then washed twice with W5 solution (Table S1). Finally, after centrifugation (100 × g, 5 min, room temperature), wild-type protoplasts were resuspended in 1 mL W1 solution (Table S1) for nuclear localization analysis, and transgenic protoplasts were cultivated as described above. The nuclear localization of Cas9-GFP in transfected protoplasts was confirmed using a TCS SP5 AOBS confocal microscope (Leica Microsystems).

### Genomic DNA extraction

Genomic DNA was extracted from the young leaves of wild-type, control, E2 and E4 plants by homogenizing 20–30 mg of leaf tissue in 400 μL of extraction buffer (Table S1). After centrifugation (13 000 × g, 10 min, room temperature), we mixed 300 μL of the supernatant with the same volume of isopropanol and incubated for 15 min at room temperature. The sample was centrifuged (13 000 × g, 15 min, room temperature) and the pellet was dried, resuspended in 100 μL of sterile water and stored at 4°C overnight. The next day, the sample was centrifuged (13 000 × g, 2 min, room temperature) and the supernatant was collected for ddPCR.

### Droplet digital PCR

The ddPCR reaction mix (22 μL) contained 11 μL of QX200 ddPCR EvaGreen supermix (Bio-Rad, Hercules, CA, USA), 0.22 μL each of the forward and reverse primer (10 μM), 1 μL of EcoRV (5 U/μL, Promega, Madison, WI, USA) and 4 ng of genomic DNA. The primers used to amplify the GFP gene were GFPfor (5′-GAA GTT CGA GGG CGA CAC-3′) and GFPrev (5′-CCG TCC TCC TTG AAG TCG-3′). *VviUBIQUITIN1* (VIT_16s0098g01190) was used as reference gene and the primers used were UBQfor (5′-TCT GAG GCT TCG TGG TGG TA-3′) and UBQrev (5′-TTT GGT TGC AAA GTG TTA GAG AA-3′). Each reaction began with denaturation at 95°C for 5 min followed by 40 cycles of 95°C for 30 s and 53°C for 1 min (GFP) or 55°C for 1 min (UBQ), then one stabilization cycle (4°C for 5 min and 90°C for 5 min). The reaction was held at 4°C before fluorescence data was collected on a QX200 droplet reader (Bio-Rad). We selected samples with more than 12 000 total droplets and good separation between positive and negative droplets. The copy number of the target gene was determined by comparing with the reference gene of known copy number using the following formula:

copy number of gene T = (gene T concentration / gene R concentration) * copy number of gene R.

where the concentrations of gene T (target gene *GFP*) and gene R (reference gene *UBQ*) were calculated using QuantaSoft software (Bio-Rad) with default settings.

### Western blotting and fluorescence assay

Total protein was extracted from the leaves of wild-type, control, E2 and E4 plants (Wu *et al*., 2014) and quantified using Bradford reagent (Sigma-Aldrich). Subsequently, 6 μg of total protein was fractionated by sodium dodecylsulfate polyacrylamide gel electrophoresis (SDS-PAGE) and transferred to a nitrocellulose membrane using the Trans-Blot *Turbo* RTA Transfer Kit (Bio-Rad). The membrane was blocked for 2 h at room temperature with 4% non-fat milk powder in PBS before incubation with the primary anti-GFP polyclonal antibody ab290 (Abcam, Cambridge, UK) diluted 1:10000, overnight at 4°C. The membrane was washed (3× 10 min in blocking solution plus 0.1% Tween-20) then incubated for 2 h at room temperature with the secondary anti-rabbit antibody (Sigma-Aldrich) diluted 1:10000. After further washes as above, the chemiluminescent signal was detected using the ECL Western Blotting substrate (Promega). We also analysed 20 μg of total protein from each sample (E2 and E4) for GFP fluorescence using a Tecan Infinite M200 PLEX instrument with 96 flat-well chimney black non-sterile plates (Thermo Fisher Scientific).

## Acknowledgements

The authors would like to thank Dr. Chiara Foresti for her kind support with the western blots and fluorescence assays. This research was funded by the European Union’s Horizon 2020 research and innovation programme under Marie Skłodowska-Curie grant no. 754345 awarded to SN, the University of Verona in the framework of the Grant Ricerca di Base “Definition of master regulator genes of fruit ripening in grapevine” awarded to SZ, and the Ministero delle Politiche Agricole Alimentari e Forestali (Mipaaf) in the framework of the BIOTECH-VITECH (CIG: 8704614AB4) project awarded to SZ.

## Author contributions

S.N., E.B. and S.Z. designed the research; S.N. performed the research and analyzed the data; E.B contributed to data analysis; E.D., M.F., S.N. and S.Z. wrote the paper.

## Data availability

The authors confirm that all the experimental data are available and accessible via the main text and/or the supplementary information.

## Conflict of interests

The authors declare no conflict of interest.

## Supplementary data

Supplementary data is available at *Horticulture Research* online.

## Supplementary Material

Web_Material_uhac240
