# Supplementary material for: DNA-free genome editing in grapevine using CRISPR/Cas9 ribonucleoprotein complexes followed by protoplast regeneration
Source: Hortic Res. 2022 Oct 26;10(1):uhac240. doi: 10.1093/hr/uhac240 (PMC10108004; doi:10.1093/hr/uhac240)
Supplement: Web_Material_uhac240 [file web_material_uhac240.zip › Supplementary Information.pdf]

Supplementary Information

DNA-free genome editing in grapevine using CRISPR/Cas9 ribonucleoprotein complexes followed by protoplast regeneration

Samaneh Najafi, Edoardo Bertini, Erica D'Incà, Marianna Fasoli, Sara Zenoni*

Department of Biotechnology, University of Verona, 37134 Verona, Italy

* Corresponding author: sara.zenoni@univr.it

Authors e-mail: samaneah.najafi@univr.it, edoardo.bertini@univr.it, erica.dinca@univr.it, marianna.fasoli@univr.it, sara.zenoni@univr.it

Table S1. Media composition

Medium	Composition
**NB2**	Nitsh's macro and microelements, MS vitamins, 0.1 g/L myo-inositol, 1 µM 6-benzylaminopurine (BAP), 5 µM 2,4-dichlorophenoxyacetic acid (2,4-D), 20 g/L sucrose, pH 6.0
**C1P**	MS macro and microelements, vitamins and amino acids as described by Torregrosa (1998), 1 g/L casein hydrolysate, 1 µM BAP, 5 µM 2,4-D, 1× Fe-EDTA, and 30 g/L sucrose
**X6**	MS salts lacking glycine and modified to contain 3.033 g/L KNO3 and 0.364 g/L NH4Cl as nitrogen sources, and 60.0 g/L sucrose, 1.0 g/L myo-inositol, 7.0 g/L TC agar and 0.5 g/L washed activated charcoal
**DMcc**	DM medium supplemented with 200 mg/L each of cefotaxime and carbenicillin
**DMcck50**	DMcc + 50 mg/L kanamycin
**Selective DM**	DMcc + 100 mg/L kanamycin
**DKW**	DKW salts supplemented with 0.3 g/L KNO3, 1.0 g/L myo-inositol, 2.0 g/L each of thiamine–HCl and glycine, 1.0 mg/L nicotinic acid, 30.0 g/L sucrose, 5.0 µM BAP, 2.5 µM each of 1-naphthaleneacetic acid (NAA) and 2,4-D, and 7.0 g/L agar TC (pH 5.7, adjusted with 1 M KOH)
**$C1^P$cck70**	$C1^P$ supplemented with 200 mg/L each of cefotaxime and carbenicillin, and 70 mg/L kanamycin
**Digestion solution**	2% w/v Cellulase Onozuka, 1% w/v Macerozyme R-10, 0.05% w/v Pectolyase Y-23, 10 mM CaCl2, 5 mM 2-(N-morpholino)ethanesulfonic acid (MES) and 0.5 M mannitol, pH 5.7
**DC Nitsch's medium**	Nitsch's macro and microelements supplemented with 2 mg/L 1NAA, 0.5 mg/L BAP, 0.3 M glucose, 0.09 M sucrose and 2 g/L gellan gum (pH 5.7)
**C2D-4B**	C2D medium supplemented with 30 g/L sucrose and 4 µM BAP, pH 5.8
**MG1**	NN macroelements, MS microelements, 1× Fe-EDTA, 1× B5 vitamins, 30 g/L sucrose, 2.5 g/L activated charcoal, pH 5.7
**MSN**	MS medium containing 30 g/L sucrose, 0.5 µM NAA and 7g/L TC agar, pH 5.8
**RIM**	MS macro and microelements, 1× Fe-EDTA, 1× T vitamins, 0.5 µM NAA, 30 g/L sucrose and 7 g/L TC agar, pH 6.0
**MMG**	0.4 M mannitol, 15 mM MgCl2, 4 mM MES, pH 5.7
**W5 solution**	2 mM MES pH 5.7, 154 mM NaCl, 125 mM CaCl2 and 5 mM KCl
**W1 solution**	0.5 M mannitol, 20 mM KCl, 4 mM MES, pH 5.7
**Extraction buffer**	200 mM Tris-HCl pH 8.0, 250 mM NaCl, 1% (w/v) SDS, 25 mM EDTA and 10 mM β-mercaptoethanol

Table S2 Depiction of the main experimental procedure differences in protoplasts isolation, transfection and plant regeneration.

**Step of the experimental procedure**	**Scintilla et al., 2021**	**This work**
Protoplasts isolation *-digestion solution-*	1% (w/v) cellulase<br>0.2% (w/v) hemicellulose<br>0.3% (w/v) macerozyme<br>in Gamborg B5<br>0.45 M mannitol	2% (w/v) cellulase<br>1% (w/v) macerozyme<br>0.05% (w/v) pectolyase<br>in MES<br>0.5 M mannitol
Protoplasts isolation *-incubation time-*	16 h	5-6 h
Protoplasts purification by sucrose gradient	yes	no
RNP preparation and Protoplast transfection	Malnoy et al., 2016	Incubation of RNP components at room temperature in the dark prior PEG transfection (Woo et al., 2015 and Osakabe et al., 2018)
Protoplast cultivation *-protoplasts concentration-*	not defined	1 x 10^5 protoplasts/mL
Protoplast cultivation *-method -*	Alginate disks	Disc-culture method
Protoplast cultivation *-medium-*	NN medium including vitamins, 88 mM sucrose, 300mM glucose, 1g/L charcoal, 0.93 µM kinetin, 2.22 µM 6-BAP, 10.7 µM NAA	NN medium including vitamins, 2mg/L NAA, 0.5 mg/L BAP, 0.3 M glucose, 0.09 M sucrose (2g/L gellan gum for solid and 0.3% activated charcoal for liquid)
Somatic embryos formation	Disks transferred in GS1CA with glutathione	Gellan gum disks with Nitsch's medium
Somatic embryos germination	NN solid medium<br>16/8 light/dark	NN solid medium<br>dark
Shoot formation		Four types of media
Plant development		Two types of media

Figure S1. Plantlets regenerated *in vitro* in different shooting media from transgenic protoplasts overexpressing GFP. (a) MG1 and (b) C2D. For each medium, we added a combination with 6-benzylaminopurine (BAP). In total we tested (a) C2D (C2D-B), C2D plus 4µM BAP (CSD+4B), (b) MG1 (MG1-B) and MG1 plus 10 µM BAP (MG1+10B) media.

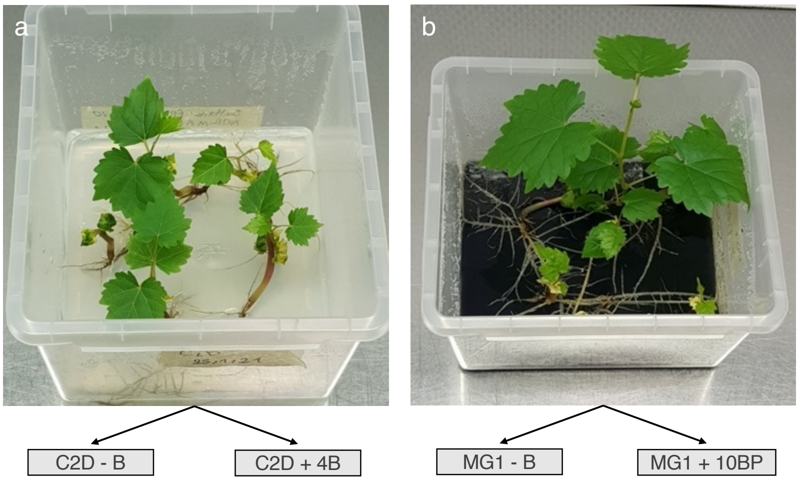

Figure S2. PCR analysis of the *GFP* gene (product = 720 bp) in plants regenerated from transgenic protoplasts overexpressing GFP. L, 100 bp ladder; –, negative control; +, positive control (pEGB3α1-TNOS::NPTII::PNOS-SF-35S::GFP::TNOS-SF); P, regenerated plant from GFP-overexpressing cv. Thompson Seedless protoplasts.

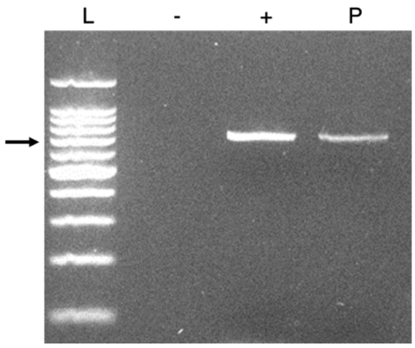

Figure S3. Direct delivery of Cas9-GFP complexes to protoplasts. Two examples of protoplasts 1 h after transfection under white light (Bright field), and UV light (GFP). The Fiji software has been used to analyze the photos and calculate the percentage of transfection. The 17% is an average of 5 analyzed photos. Bars = 5 μm.

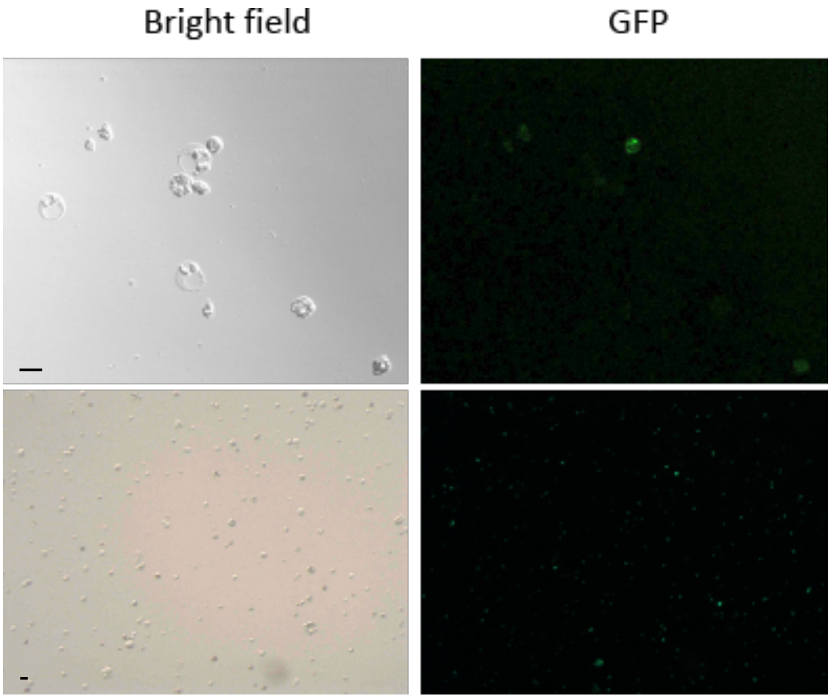

Figure S4. Sequences of the sgRNA target sites.

a

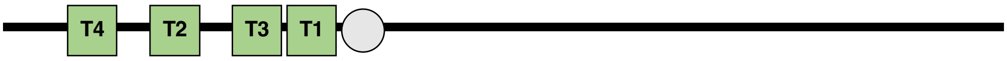

b

Target	Target sequence (5'-3')	GC%	Number of putative off-target sites
T1	CCCACCCTCGTGACCACCTTC	61.1	0
T2	CGAGGGCGACGCCACCTACGG	72.2	0
T3	CCGGCAAGCTGCCCGTGCCCT	71.1	0
T4	CCTGGTCGAGCTGGACGGCGA	72.2	0

Figure S5. Droplet digital PCR amplitude plot. The set threshold is shown as a pink line, above which are positive droplets (blue) containing at least one copy of the target DNA and below which are negative droplets (gray) lacking the target DNA. The ddPCR reactions are divided by the vertical dotted yellow line. -, negative control; WT, wild-type plant; C, regenerated plant from only PEG-transfected protoplasts; E2, regenerated plant from transfected protoplasts with RNP2 not overexpressing GFP; E4, from transfected protoplasts with RNP4 not overexpressing GFP; GFP, target gene; UBQ, *VviUBIQUITIN1* (VIT_16s0098g01190) was used as reference gene. Specific values are reported in the table below the plot.

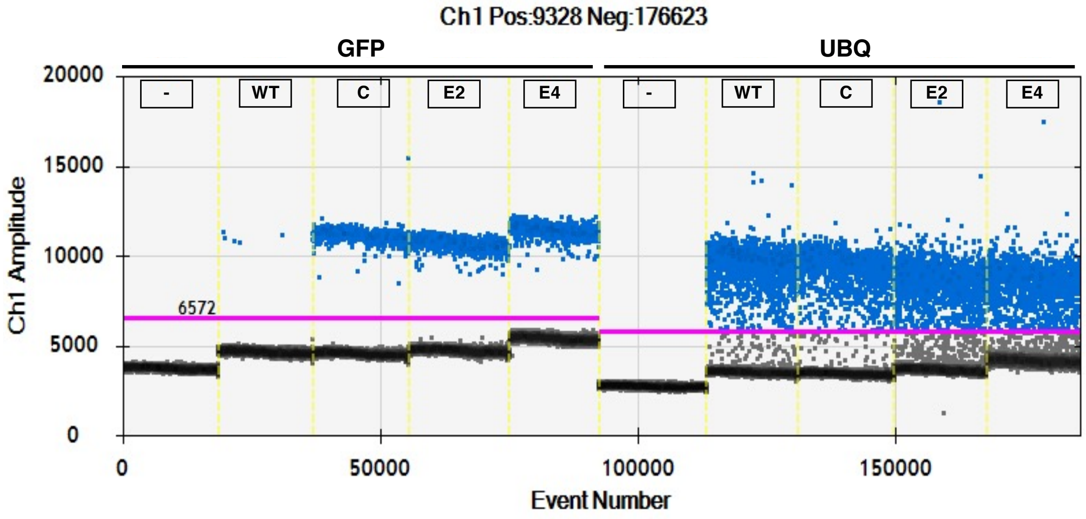

Gene	Sample	GFP concentration	UBQ concentration	GFP copy number
	-	0,00	0,00	
	WT	7,04	1520,20	0,00
**GFP**	C	1267,20	997,70	1,27
	E2	1478,40	1169,30	1,26
	E4	1696,20	1123,10	1,51
	-	0,00	0,00	
	WT	3040,40	1520,20	
**UBQ**	C	1995,40	997,70	
	E2	2338,60	1169,30	
	E4	2246,20	1123,10	
